# Computed tomography and histopathological findings after embolization with inherently radiopaque 40μm-microspheres, standard 40μm-microspheres and iodized oil in a porcine liver model

**DOI:** 10.1371/journal.pone.0198911

**Published:** 2018-07-09

**Authors:** Dominik F. Vollherbst, Theresa Gockner, Thuy Do, Kerstin Holzer, Carolin Mogler, Paul Flechsig, Alexander Harms, Christopher L. Schlett, Philippe L. Pereira, Götz M. Richter, Hans U. Kauczor, Christof M. Sommer

**Affiliations:** 1 Department of Neuroradiology, University Hospital Heidelberg, Heidelberg, Germany; 2 Clinic for Diagnostic and Interventional Radiology, University Hospital Heidelberg, Heidelberg, Germany; 3 Clinic for Diagnostic and Interventional Radiology, University Hospital Mainz, Mainz, Germany; 4 Department of General Pathology, University Hospital Heidelberg, Heidelberg, Germany; 5 Institute of Pathology, Technical University Munich, Munich, Germany; 6 Department of Nuclear Medicine, University Hospital Heidelberg, Heidelberg, Germany; 7 Clinic for Radiology, Minimally-invasive Therapies and Nuclear Medicine, SLK Kliniken Heilbronn GmbH, Heilbronn, Germany; 8 Clinic for Diagnostic and Interventional Radiology, Klinikum Stuttgart, Stuttgart, Germany; Vanderbilt University Medical Center, UNITED STATES

## Abstract

**Purpose:**

The present study compared standard computed tomography (CT) and histopathological findings after endovascular embolization using a prototype of inherently radiopaque 40μm-microspheres with both standard 40μm-microspheres and iodized oil in a porcine liver model.

**Materials and methods:**

Twelve pigs were divided into six study groups, of two pigs each. Four pigs were embolized with iodized oil alone and four with radiopaque microspheres; two animals in each group were sacrificed at 2 hours and two at 7 days. Two pigs were embolized with radiopaque microspheres and heparin and sacrificed at 7 days. Two pigs were embolized with standard microspheres and sacrificed at 2 hours. CT was performed before and after segmental embolization and before sacrifice at 7 days. The distribution of embolic agent, inflammatory response and tissue necrosis were assessed histopathologically.

**Results:**

Radiopaque microspheres and iodized oil were visible on standard CT 2 hours and 7 days after embolization, showing qualitatively comparable arterial and parenchymal enhancement. Quantitatively, the enhancement was more intense for iodized oil. Standard microspheres, delivered without contrast, were not visible by imaging. Radiopaque and standard microspheres similarly occluded subsegmental and interlobular arteries and, to a lesser extent, sinusoids. Iodized oil resulted in the deepest penetration into sinusoids. Necrosis was always observed after embolization with microspheres, but never after embolization with iodized oil. The inflammatory response was mild to moderate for microspheres and moderate to severe for iodized oil.

**Conclusion:**

Radiopaque 40μm-microspheres are visible on standard CT with qualitatively similar but quantitatively less intense enhancement compared to iodized oil, and with a tendency towards less of an inflammatory reaction than iodized oil. These microspheres also result in tissue necrosis, which was not observed after embolization with iodized oil. Both radiopaque and standard 40μm-microspheres are found within subsegmental and interlobar arteries, as well as in hepatic sinusoids.

## Introduction

Minimally invasive transcatheter embolotherapy is an established treatment for benign and malignant hypervascular tumors. In the liver, transarterial embolization is an evidence-based first-line treatment for patients with intermediate stage hepatocellular carcinoma and a second-line treatment for patients with liver-dominant metastases [1, 2]. A broad range of different embolic agents are commercially available for liver tumor embolization [2, 3]. However, no consensus is available on the specific type of embolic material that is superior for the different tumor entities regarding oncological efficacy, complication rate, and biocompatibility [2, 4, 5].

An important issue for effective tumor embolization is the embolic agent being capable of reaching the tumor vasculature for complete devascularization and the chemotherapeutic agent being distributed homogeneously throughout the target volume [2, 3, 6]. Generally, the smaller the diameter of a particulate embolic agent, the deeper its penetration into the tumor and the greater the treatment effect [3, 7]. In this context, embolization with the smallest available embolic agents, such as iodized oil (Lipiodol Ultra-Fluide; Guerbet, Roissy, France) and microspheres (e.g., Embozene 40 [CeloNova BioSciences, San Antonio, USA/Boston Scientific, Marlborough, USA]) bear the risk of complications, such as bile duct injury due to occlusion of the peribilliary plexus, non-target embolization due to arterio-venous shunts or occult arterial collateralization, and fulminant tumor necrosis with subsequent embolism of necrotic tissue [2, 8, 9]. In order to obtain a controllable level of distal arterial occlusion, narrow-size calibrated small microspheres with a defined and uniform diameter of ≤75μm are increasingly being used [6, 7, 10]. Embozene 40 and Tandem 40 microspheres (CeloNova BioSciences, San Antonio, USA/Boston Scientific, Marlborough, USA) are currently the smallest commercially available permanent microspheres that are clinically used for transarterial embolization [2, 8, 11].

The introduction of inherently radiopaque, x-ray-visible microspheres is another trend in liver tumor embolization [12–16]. Currently, the commercially available Embozene microspheres are not inherently visible applying standard x-ray or magnetic resonance imaging (MRI) techniques. In contrast, iodized oil, one of the first embolic agents used for transarterial embolization of liver tumors, features excellent x-ray visibility applying fluoroscopy, radiography, or computed tomography (CT), and can still be regarded as a standard for transarterial tumor embolization in the liver [17, 18].

Animal models have been shown to be suitable for preclinical evaluation of the characteristics of embolic agents, such as visibility, vascular distribution, and effects on the treated tissue [7, 10–13].

The aim of the present study was to compare a prototype of narrow-size calibrated radiopaque 40μm-microspheres with iodized oil and narrow-size calibrated non-radiopaque standard 40μm-microspheres as embolic agents with particularly deep penetration into the liver applying standard CT and histopathology.

## Materials and methods

State Animal Care and Ethics Committee approval was obtained (Regional Council Karlsruhe, Germany; permit number: 35–9185.81/G-17/15). All experiments were performed in accordance with the Guide for the Care and Use of Laboratory Animals.

### Study groups

Twelve animals were equally and randomly divided into six study groups (Table 1) according to type and suspension of embolic agent and survival time: iodized oil, acute setting (IO.a), iodized oil, subacute setting (IO.sa), radiopaque microspheres in suspension with saline, acute setting (RMS.a), radiopaque microspheres in suspension with saline and iodinated contrast material, subacute setting (RMS.sa), radiopaque microspheres in suspension with saline, iodinated contrast material and heparin, subacute setting (RMS-HEP.sa) and standard microspheres in suspension with saline, acute setting (SMS.a). Commercially available iodized oil was used as the pure embolic agent. The commercial product Embozene 40 (CeloNova BioSciences, San Antonio, USA/Boston Scientific, Marlborough, USA) was used as the standard microspheres, consisting of narrow-size calibrated microspheres with a hydrogel core and a Polyzene-F shell with a mean diameter of 40±10 μm [19, 20]. There have been previous preclinical and clinical experiences with these microspheres, including long-term follow-up; thus, no special survival experiments (subacute setting) were performed for this embolic agent in order to reduce the number of animals required [8, 11, 19]. A prototype of Embozene 40 (CeloNova BioSciences, San Antonio, USA/Boston Scientific, Marlborough, USA) with the addition of barium sulfate as the radiopaque material was used as the radiopaque microspheres.

**Table 1:** Study groups.

Study group	Embolic agent	Experimental setting / survival time	Embolic mixture / preparation of the embolic agent	Embolization endpoint	Injected volume of the embolic mixture (Pig A / Pig B)
IO.a	Iodized oil^1^	Acute setting / 2 hours	Pure	Defined volume of embolic agent	0.5 mL / 0.5 mL
IO.sa		Subacute setting / 7 days	Pure	Peripheral stasis with remaining blood flow in the central artery	1.0 mL / 1.5 mL
RMS.a	Radiopaque microspheres^2^	Acute setting / 2 hours	1 mL embolic agent in suspension with 10 mL 0.9% saline	Defined volume of embolic agent	5.5 mL / 5.5 mL
RMS.sa		Subacute setting / 7 days	1 mL embolic agent in suspension with 2 mL saline and 8 mL iodinated contrast agent	Peripheral stasis with remaining blood flow in the central artery	5.5 mL / 7.7 mL
RMS-HEP.sa			1 mL embolic agent in suspension with 2 mL 0.9% saline, 8 mL iodinated contrast material and 2000 IU heparin		8.8 mL / 13.2 mL
SMS.a	Standard microspheres^3^	Acute setting / 2 hours	1 mL embolic agent in suspension with 10 mL 0.9% saline	Defined volume of embolic agent	5.5 mL / 5.5 mL

^1^Lipiodol Ultra-Fluide (Guerbet, Roissy, France)

^2^Prototype of inherently radiopaque 40μm-microspheres (CeloNova BioSciences, San Antonio, USA / Boston Scientific, Marlborough, USA)

^3^Embozene 40 microspheres (CeloNova BioSciences, San Antonio, USA / Boston Scientific, Marlborough, USA)

IO.a: Iodized oil, acute setting; IO.sa: Iodized oil, subacute setting, RMS.a: Radiopaque microspheres, acute setting; RMS.sa: Radiopaque microspheres, subacute setting; RMS-HEP.sa: Radiopaque microspheres with addition of heparine, subacute setting, SMS.a: Standard microspheres, acute setting; IU: International Units.

For the acute setting, a defined volume of radiopaque and standard microspheres suspended in saline, without addition of iodinated contrast material, was used to assess whether microspheres are inherently visible applying standard CT. A defined volume of iodized oil was used as a control. We selected this specific embolization technique for two reasons. First, 0.5 mL of embolic agent has been reported to be sufficient for the occlusion of subsegmental arteries in the same species [20, 21]. Second, hyperdense areas diagnosed by CT correspond to enhancement induced by the embolic agent itself, and not by iodinated contrast material [20, 21]. For the subacute setting, the embolization endpoint was defined as in clinical settings: stasis within the embolized liver segment but persistence of blood flow within the feeding central liver artery determined by angiography 5 minutes after embolization. For the subacute setting, iodinated contrast material was added to the microspheres as is standard in clinical practice [1–3]. The addition of iodinated contrast material to the microspheres results in a homogeneous suspension, which is effective for homogeneous delivery of the microspheres into the microcatheter and obtaining optimal rheological properties for flow-directed embolization [3, 22]. A suspension consisting of inherently visible microspheres, normal saline, and iodinated contrast material was used in the latest studies on radiopaque microspheres [14, 23]. Accordingly, the visibility of the embolic agent may be increased at 2 hours by iodinated contrast material, however during 7 days of follow-up, the iodinated contrast material washes out; thus the visibility at 7 days is expected to be attributed to the radiopaque microspheres exclusively [13, 24, 25]. The rationale for adding heparin (study group RMS-HEP.sa) was to reduce the formation of coagulation thrombus within the target vessel during embolization, i.e., to obtain arterial occlusion that is mainly caused by microspheres, and not by an additional coagulation thrombus component, with a potential for recanalization over time, and therefore unpredictable embolization results.

### Animal procedure and embolization technique

German Landrace pigs (body weight 33–37 kg) were used. The survival time was 2 hours (acute setting; n = 6) or 7 days (subacute setting; n = 6). Before the first intervention, the animals were fasted overnight. For the subacute setting, during the first 12 hours after intervention, animals only received water before food and liquid were made available ad libitum. The animals were followed, with daily assessment of activity, pain, dietary intake, and urine and fecal output. Analgesia was administered daily using carprofen (2 mg/kg; Rimadyl, Pfizer Animal Health, New York, USA). The animal housing environment (cage size of 3 m^2^ per animal, individual housing) was maintained at a temperature of 18 C° to 22°C at 60% to 80% humidity with a 12-hour light-dark cycle. All interventions were performed under deep general anesthesia. Anesthesia was induced with an intravenous injection of ketamin (10 mg/kg; Ketamin, Medistar, Hannover, Germany), midazolam (0.4 mg/kg; Dormicum, Roche, Basel, Switzerland) and azaperone (6 mg/kg; Stresnil, Janssen Animal Health, Beerse, Belgium) and maintained with repetitive intravenous injections of ketamine and midazolam (dosed according to the effect). After cutdown, a 4F introducer sheath was inserted in the femoral artery. After positioning a 4F Cobra catheter in the main hepatic artery, a 2.8F microcatheter (Progreat; Terumo, Tokyo, Japan) was coaxially positioned in a segmental artery. In each animal, one segmental artery (either of the right or left liver lobe) was embolized. Pre-embolization angiograms were performed for treatment planning. Post-embolization angiograms were only performed for the subacute setting in order to confirm the embolization endpoint as described above. Before, 2 hours after or 2 hours and 7 days after embolization, a non-enhanced CT was applied with a standard scanner (Somatom Definition Flash; Siemens Medical Solutions, Forchheim, Germany). CT data were acquired according to clinical practice using an automated dose optimization software (CareDose and CarekV; Siemens Medical Solutions, Forchheim, Germany; dose setting for CTDI_vol_: 6.0 mGy) and a collimation of 64x0.6 mm. CT images were reconstructed as multi-planar images by applying an iterative reconstruction software (Admire 3/5; Siemens Medical Solutions, Forchheim, Germany; slice thickness: 3/2 mm, reconstruction kernel: I30-30). The animals were sacrificed with an intravenous injection of 20 mL potassium chloride 7.5%. After sacrifice livers were harvested and tissue from embolized and non-embolized lobes preserved in a solution of 4% formalin. In each liver, 10 tissue fractions of the embolized liver segments were paraffin-embedded. The embolized liver segments were located by the macroscopic signs of embolization (e.g. tissue necrosis) and/or by macroscopic landmarks, defined under consideration of the pre-embolization angiograms and the post-embolization CT images.

### Histopathology

For the histopathological work-up, 4μm sections were obtained from the paraffin-embedded specimens. Hematoxylin and eosin (HE), Elastica van Gieson (EvG), and Masson Golder (MG) staining was performed. For direct visualization of iodized oil, Sudan III staining was performed, which specifically stains lipids [26].

### Study goals

The visibility of the embolic agents on CT was assessed 2 hours (acute and subacute setting) and 7 days after embolization (subacute setting only) by D.F.V. and C.M.S. with 5 and 10 years of experience in radiology, respectively. The enhancement pattern was described qualitatively and compared quantitatively between the different study groups. The vascular distribution pattern of the embolic agents by applying histopathology was described. Inflammation, necrosis and other conspicuities of the embolized liver tissue were assessed.

## Results

All animals were treated as planned. The six animals with the subacute setting ate and gained weight as expected. No complications occurred during the embolization procedure, and there were no adverse events or signs of toxicity during follow-up.

### Standard CT findings

**Fig 1:**
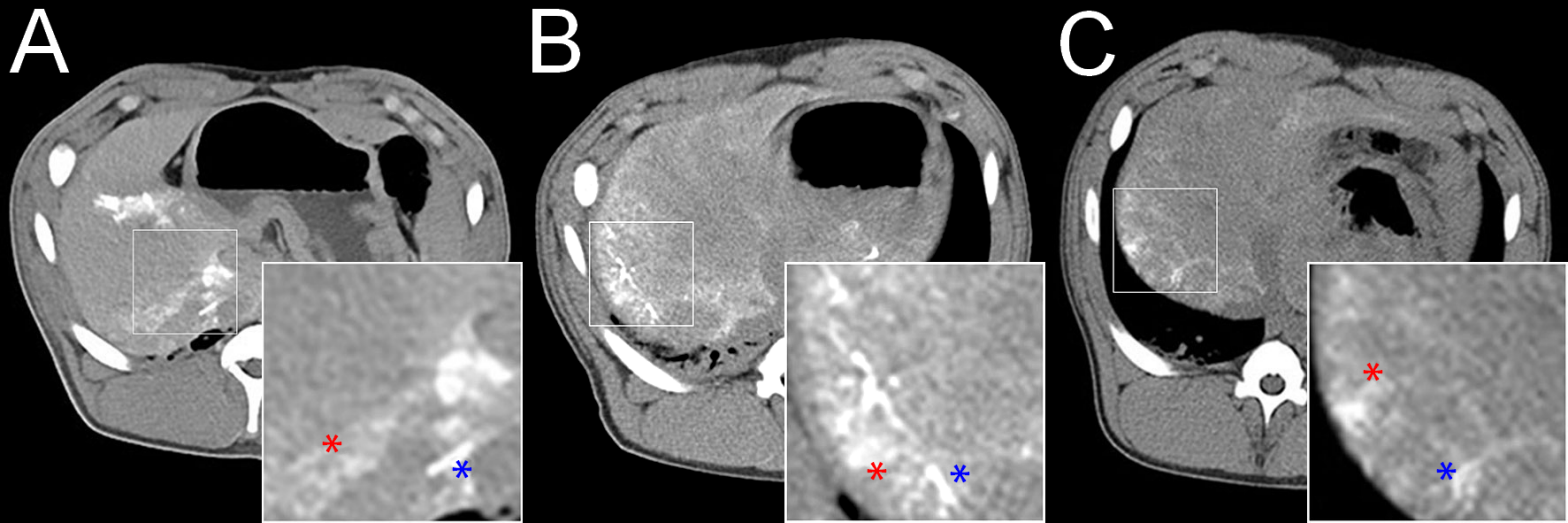
Iodized oil–standard CT findings. **A** IO.a. Non-enhanced CT 2 hours after embolization with 0.5 mL iodized oil. Linear hyperdensities (blue asterisk) corresponding to an occluded subsegmental artery (arterial enhancement) and patchy hyperdensities (red asterisk) corresponding to occluded interlobular arteries and sinusoids (parenchymal enhancement). **B** IO.sa. Non-enhanced CT 2 hours after embolization. Note the lower arterial (blue asterisk) and parenchymal (red asterisk) enhancement compared to A. **C** IO.sa. Non-enhanced CT 7 days after embolization. Note the less intense arterial (blue asterisk) and parenchymal (red asterisk) enhancement compared to B.

**Fig 2:**
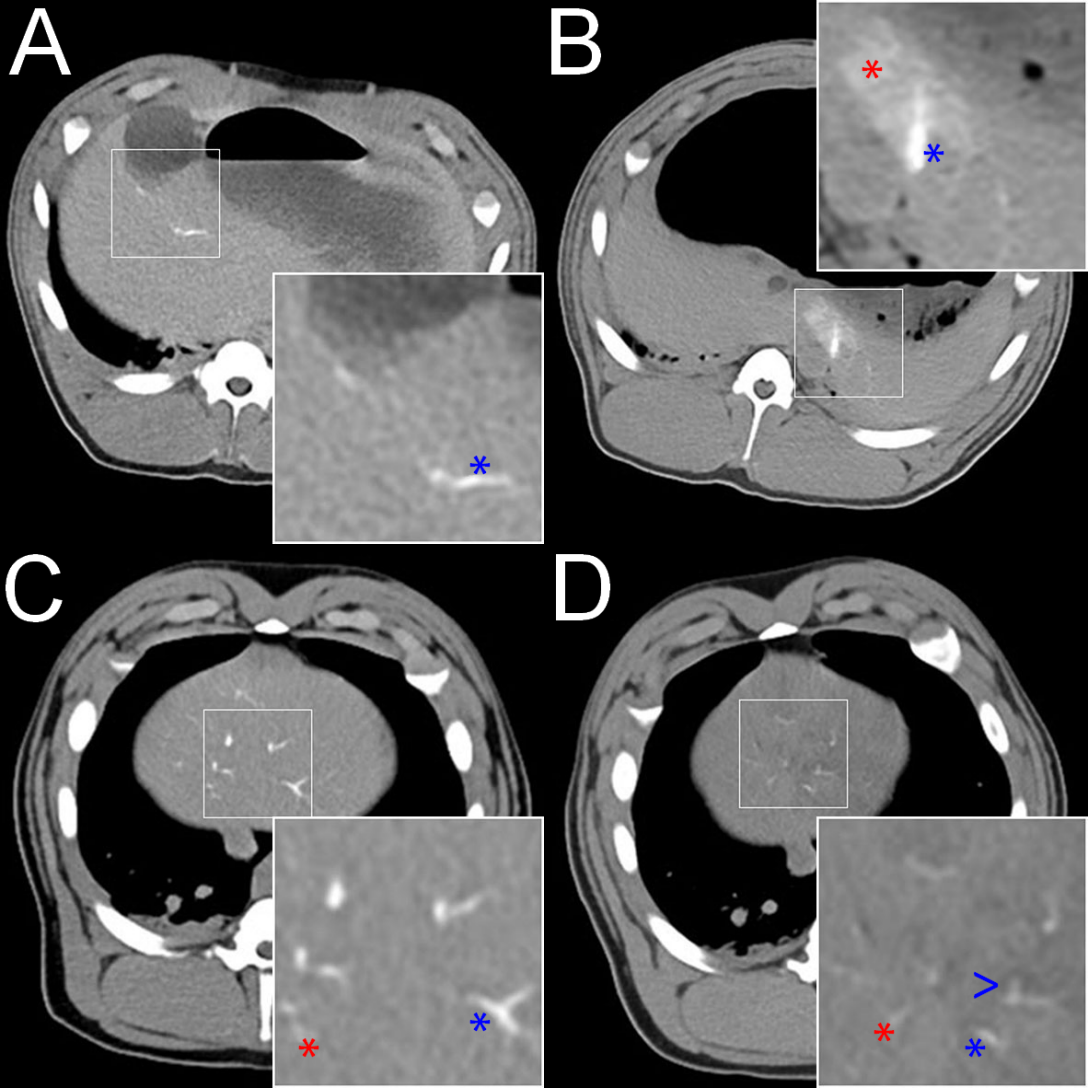
Radiopaque microspheres–standard CT findings. **A** RMS.a. Non-enhanced CT 2 hours after embolization. Marked linear hyperdensity (blue asterisk) corresponding to an occluded subsegmental artery (arterial enhancement). The type of arterial enhancement was comparable to iodized oil. **B** RMS.sa. Non-enhanced CT 2 hours after embolization. Linear (blue asterisk) and patchy hyperdensities (red asterisk) corresponding to occluded subsegmental and interlobular arteries and sinusoids (arterial and parenchymal enhancement). Note the more intense arterial and parenchymal enhancement compared to A. The type of arterial and parenchymal enhancement was comparable with iodized oil. **C** RMS-HEP.sa. Non-enhanced CT 2 hours after embolization. Note the more intense arterial (blue asterisk) and parenchymal enhancement (red asterisk) than A and B. **D** RMS-HEP.sa. Non-enhanced CT 7 days after embolization. Note the weaker arterial (blue asterisk) and parenchymal (red asterisk) enhancement compared to C. Note the hypodense areas in the embolized liver parenchyma (blue arrowhead) representing tissue necrosis.

**Fig 3:**
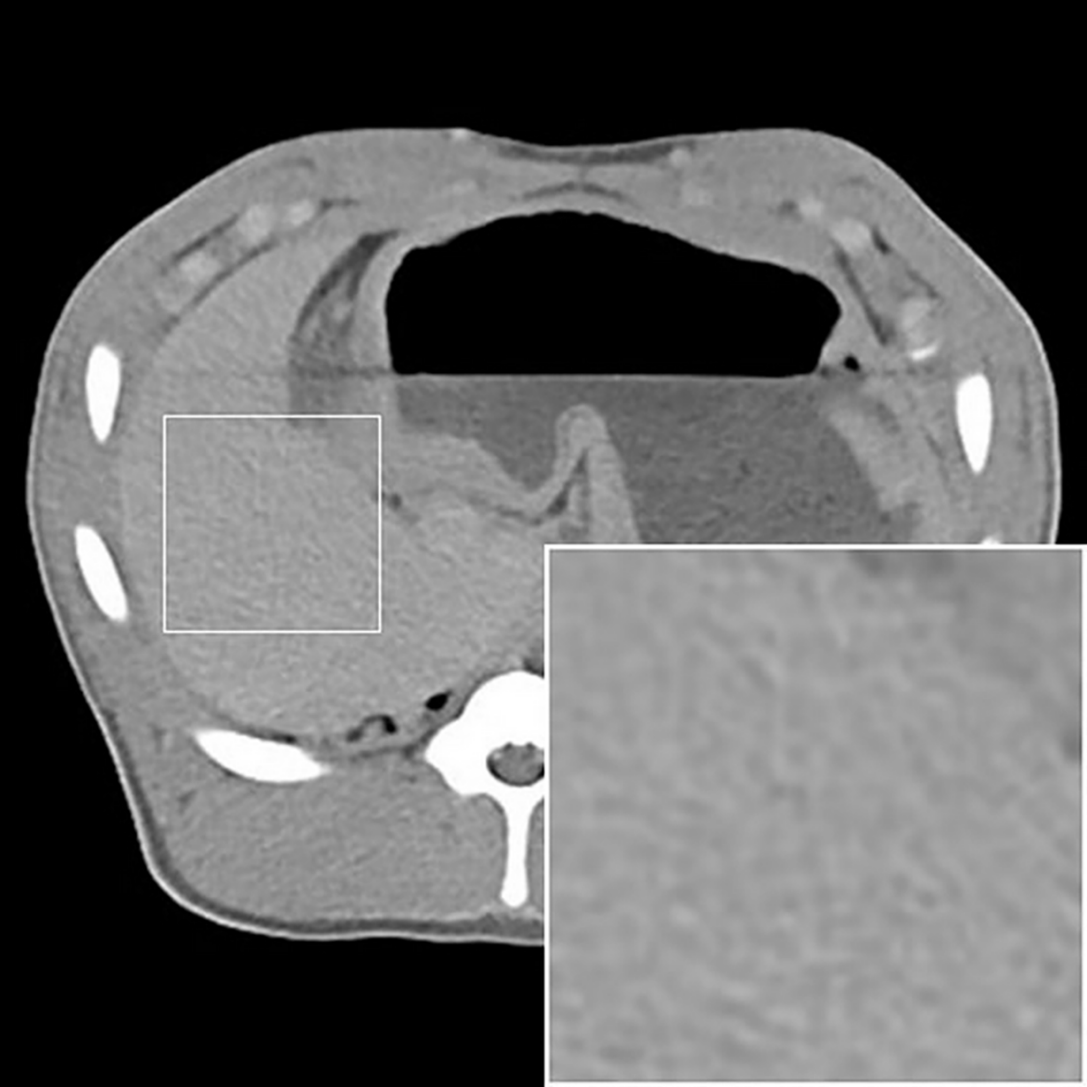
Standard microspheres–standard CT findings. SMS.a. Non-enhanced CT 2 hours after embolization. In contrast to the other study groups, there was neither arterial nor parenchymal enhancement.

CT findings are summarized in Table 2. In all animals, compared to the pre-embolization CT, a slight diffuse increase in the density of the entire liver (<15 Hounsfield Units [HU]) was observed 2 hours after embolization, which is most likely attributed to the retention iodinated contrast material after diagnostic angiography. This slight diffuse increase in the density of the entire liver was fully reversible during the 7 days of follow-up. After embolization, the embolic agents, if visible, exhibited two different enhancement patterns: linear hyperdensities, corresponding to the embolic agent occluding subsegmental and interlobular arteries (defined as arterial enhancement) and patchy hyperdensities, corresponding to the embolic agent occluding interlobular arteries and sinusoids (defined as parenchymal enhancement). For IO.a (Fig 1), intense arterial and parenchymal enhancement was observed in both animals, though with different intensity. For IO.sa, 2 hours after embolization, the same type of arterial and parenchymal enhancement was observed as for IO.a, though with a lower intensity. During the 7 day follow-up, the intensity of both arterial and parenchymal enhancement diminished in both animals. For RMS.a (Fig 2), similar to IO.a, both types of enhancement were noted in both animals, though, with a lower intensity. In addition, the intensity of arterial and parenchymal enhancement was different in both animals in this group. Two hours after embolization, the arterial and parenchymal enhancement was more intense for RMS.sa than RMS.a. Furthermore, 2 hours after embolization, there was a higher intensity of arterial enhancement and a comparable intensity of parenchymal enhancement in RMS-HEP.sa compared to RMS.sa (Fig 2). Seven days after embolization, hypodensities were present in the embolized liver parenchyma only for RMS-HEP.sa, corresponding to areas of tissue necrosis. In summary, the type of enhancement was comparable throughout all study groups with iodized oil and radiopaque microspheres, but the arterial and parenchymal enhancement was less intense for radiopaque microspheres. Furthermore, the intensity of both arterial and parenchymal enhancement diminished over the 7 days of follow-up for all subacute animals. For SMS.a, there was neither arterial nor parenchymal enhancement 2 hours after embolization (Fig 3).

**Table 2:** Standard CT findings.

Study group	Visibility of the embolic agent 2 hours after embo-lization	Arterial enhance-ment 2 hours after embo-lization	Parenchymal enhancement 2 hours after embolization	Visibility of the embolic agent 7 days after embo-lization^1^	Arterial enhancement 7 days after embollization	Parenchymal enhancement 7 days after embolization	Additional findings
(2 pigs per group)	(Pig A / Pig B)	(Pig A / Pig B)	(Pig A / Pig B)	(Pig A / Pig B)	(Pig A / Pig B)	(Pig A / Pig B)	
Pig A / Pig B	Pig A / Pig B	Pig A / Pig B	Pig A / Pig B	Pig A / Pig B	Pig A / Pig B	Pig A / Pig B	Pig A and Pig B
IO.a^2^	yes / yes	yes / yes	yes / yes	-	-	-	-
IO.sa^2^	yes / yes	yes / yes	yes / yes	yes / yes	yes / yes	yes / yes	
RMS.a	yes / yes	yes / no	yes / yes	-	-	-	-
RMS.sa	yes / yes	yes / yes	yes / no	yes / yes	yes / yes	yes / no	-
RMS-HEP.sa^3^	yes / yes	yes / yes	yes / yes	yes / yes	yes / no	yes / yes	Perivascular and parenchymal hypodensities representing necrosis within the embolized liver segments
SMS.a	no / no	no / no	no / no	-	-	-	-

Note: In all animals, 2 hours after embolization, a slight increase of the density of the liver parenchyma was observed (which can be attributed most likely to iodinated contrast material retention after diagnostic angiography)

^1^On day 7, the arterial and parenchymal enhancement was less intense for all study groups compared to the 2 hours follow-up

^2^Enhancement was more intense for iodized oil (IO.a and IO.sa) than for radiopaque microspheres (RMS.a and RMS.sa)

^3^The addition of heparin to the embolic suspension led to a more intense arterial enhancement (RMS-HEP.sa versus RMS.sa).

### Histopathological findings

**Fig 4:**
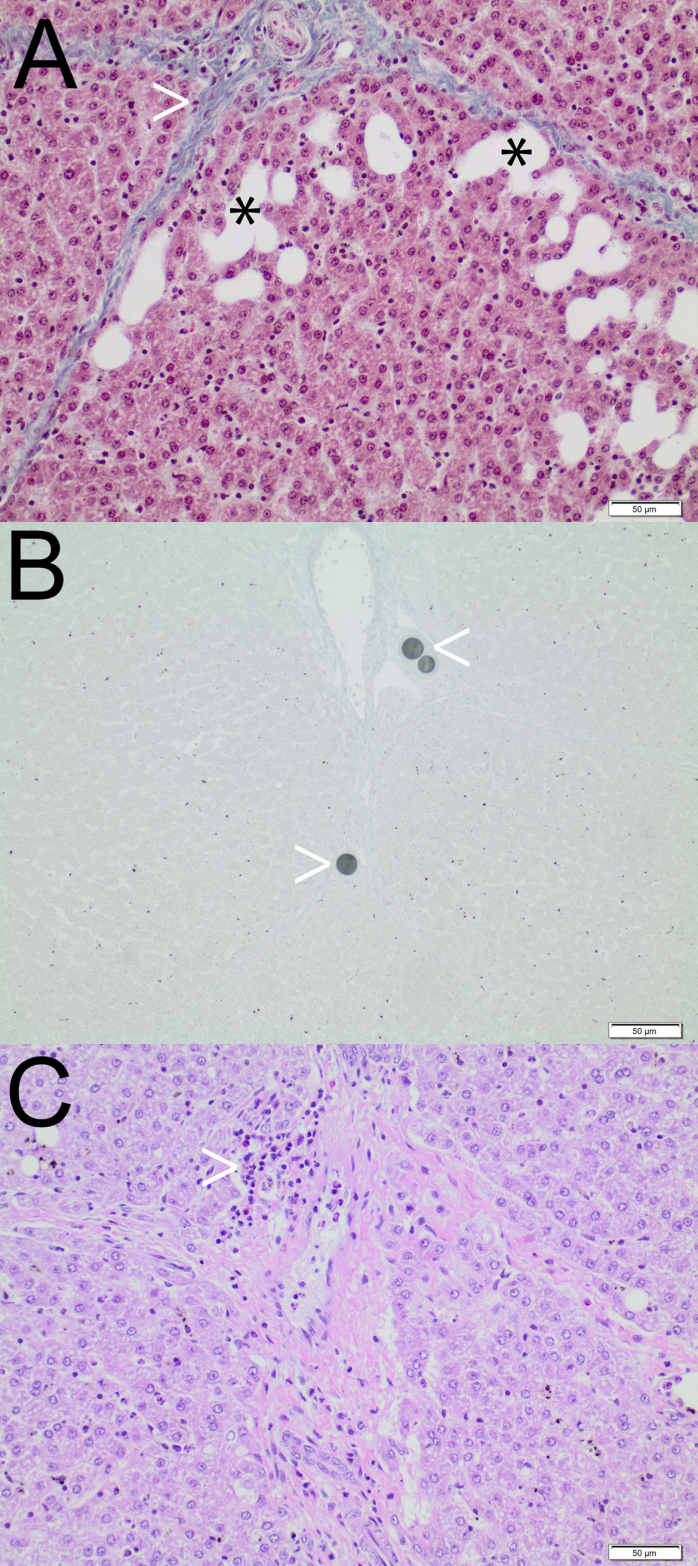
Iodized oil–histopathological findings. **A** IO.a. MG staining. Preparation artefacts due to degreasing of iodized oil during routine histopathological work-up appear as oval cavities within the sinusoids (black asterisk). Note the integrity of the liver lobule and periportal field (white arrowhead). **B** IO.sa. Sudan III staining. Iodized oil in interlobular arteries (white arrowheads). **C** IO.sa. HE staining. Infiltration of the periportal fields by lymphocytes, plasma cells and eosinophilic granulocytes (white arrowhead). Note the absence of parenchymal necrosis.

**Fig 5:**
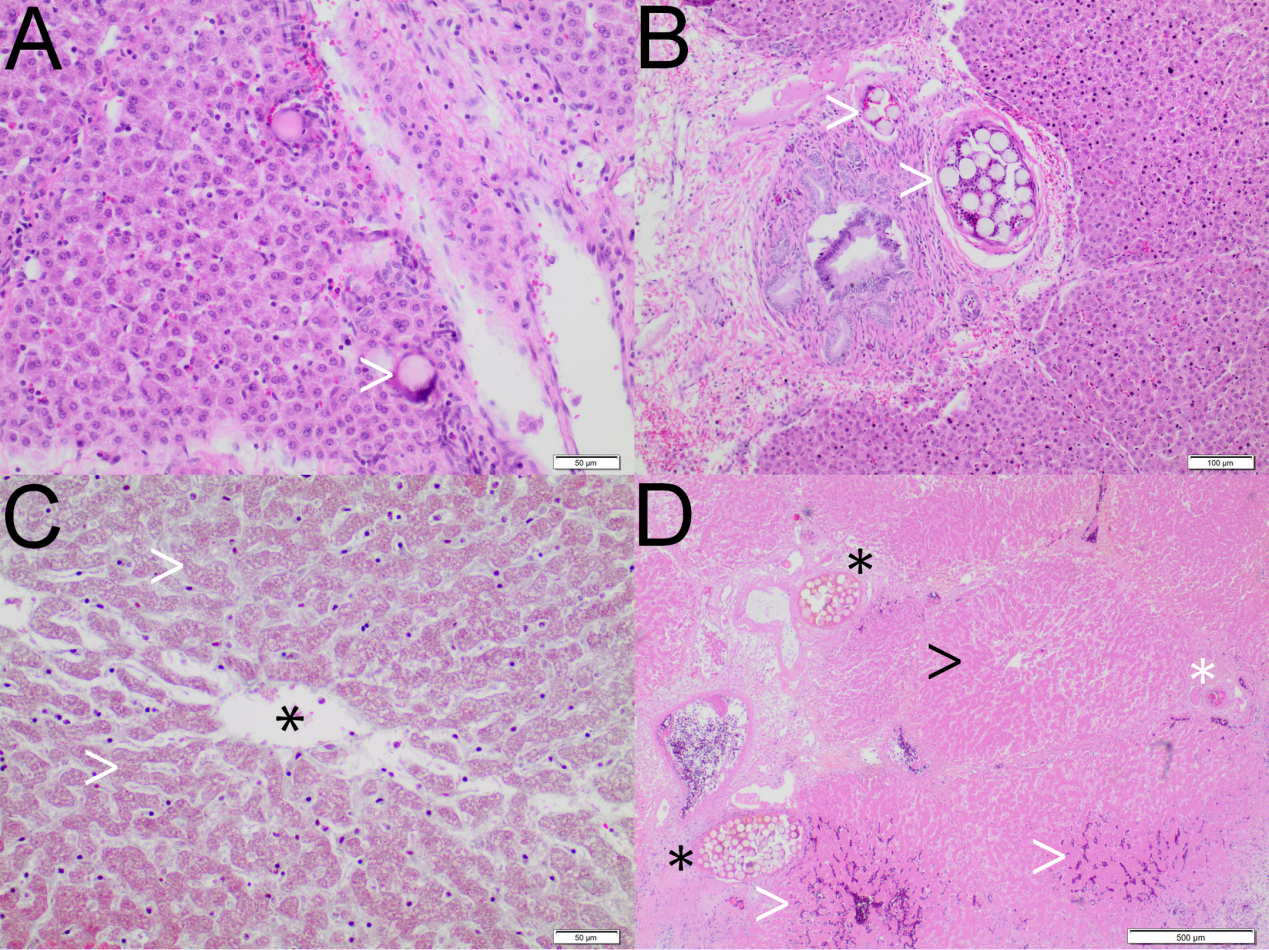
Radiopaque microspheres–histopathological findings. **A** RMS.a. HE staining. Incipient sinusoidal penetration of microspheres (white arrowhead). **B** RMS.sa. HE staining. Multiple microspheres within a subsegmental artery (white arrowheads). There were only mild inflammatory changes compared to iodized oil, but signs of cell death were present. **C** RMS.sa. HE staining. Focal areas of parenchymal necrosis, represented by anucleate hepatocytes (white arrowhead). The black asterisk indicates the central vein. **D** RMS-HEP.sa. HE staining. Seven days after embolization, there were large areas of severe confluent parenchymal necrosis (black arrowhead), accompanied by a hemorrhagic rim (white arrowheads) with necrosis of the periportal fields. Note the microspheres within the subsegmental (black asterisk) and interlobular arteries (white asterisk).

**Fig 6:**
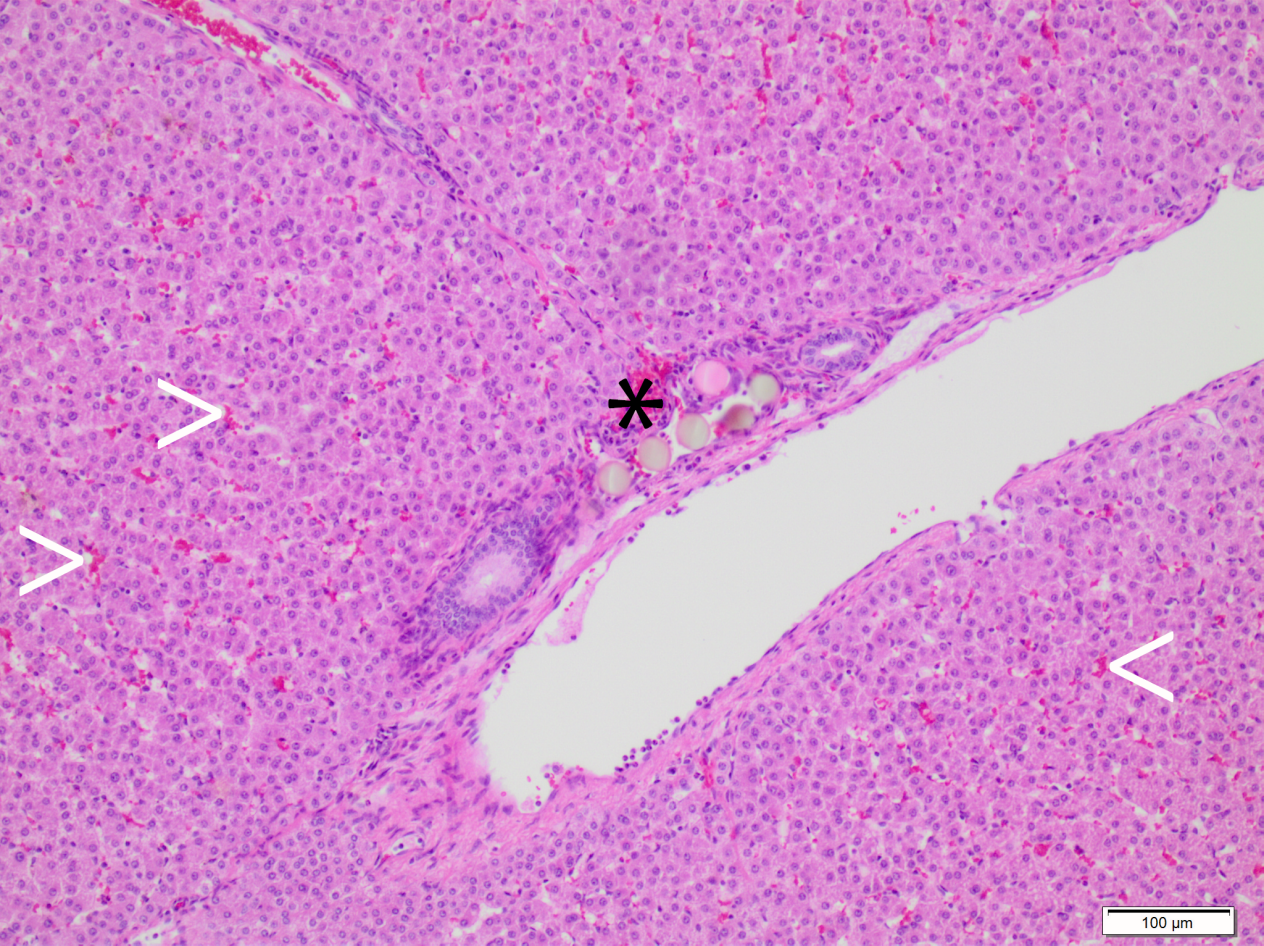
Standard microspheres–histopathological findings. SMS.a. HE staining. Microspheres in the interlobular arteries (black asterisk). Note the blood retention within the sinusoids (white arrowheads), which is a potential sign of embolization-related reactive hyperemia. There were no qualitative differences regarding the histopathological findings between the standard and the radiopaque microspheres for the acute setting.

Histopathological findings are summarized in Table 3 and Figs 4–6. For RMS.a and SMS.a, but not for IO.a, dilated sinusoids with blood retention were observed. Iodized oil was detected within sinusoids as oval cavities with 10–30 μm in size, representing iodized oil preparation artefacts (HE, MG and EvG staining), and within subsegmental and interlobular arteries as black dots approximately 30–50 μm in size (Sudan III staining). Radiopaque and standard microspheres were detected as 30 to 50 μm round globuli within subsegmental and interlobular arteries and sinusoids. Neither the morphology nor arterial distribution pattern was qualitatively different between the two types of microspheres or between RMS.sa and RMS-HEP.sa. The arterial penetration depth was higher for iodized oil than microspheres, and the arterial distribution pattern was comparable between the subacute and acute setting for iodized oil and microspheres, respectively. An inflammatory response was observed for all types of embolic agents after 7 days. For IO.sa, there was mild to moderate leukocyte infiltration of the lobules and a moderate to severe leukocyte infiltration of the periportal fields, indicating moderate to severe parenchymal and periportal inflammation. Leukocyte infiltration was also observed for RMS.sa and RMS-HEP.sa, though with lower severity, corresponding to mild to moderate inflammation. For the acute setting (IO.a, RMS.a and SMS.a) no signs of inflammation were observed. For radiopaque microspheres, different degrees of necrosis were noted for the different embolic mixtures. For RMS.sa, there were focal areas of parenchymal necrosis with vital periportal fields. For RMS-HEP.sa, large areas of confluent parenchymal necrosis were observed with a hemorrhagic rim and necrosis of the periportal fields. No necrosis was detected for IO.a, IO.sa, RMS.a or SMS.a.

**Table 3:** Histopathological findings.

Study groups	Vascular distribution of the embolic agent	Signs of inflammation	Tissue necrosis	Blood retention within the sinusoids
IO.a	Periportal and sinusoidal distribution	No	No	Mild
IO.sa		Moderate leukocyte infiltration of the lobules and multiple leukocytes (lymphocytes, plasma cells and a few eosinophilic granulocytes) within the periportal fields indicating moderate to severe parenchymal and periportal inflammation		
RMS.a	Numerous microspheres in subsegmental and interlobular arteries; only a few microspheres inside interlobular arteries and sinusoids^1^	No		Mild to moderate
RMS.sa		Few leukocytes within the lobules, some leukocytes (lymphocytes, plasma cells and a few eosinophilic granulocytes) within the periportal fields indicating mild parenchymal and periportal inflammation^2^	Focal areas of parenchymal necrosis with vital periportal fields	
RMS-HEP.sa			Large areas of severe confluent parenchymal necrosis with a hemorrhagic rim and necrosis of the periportal fields	
SMS.a		No	No	

^1^No qualitative difference in vascular distribution between SMS and RMS for the acute setting

^2^No qualitative difference in inflammation between RMS and RMS-HEP for the subacute setting.

### Discussion

In this experimental study, narrow-size calibrated, very small, inherently radiopaque microspheres were visible 2 hours and 7 days after embolization on standard CT images. In contrast, standard microspheres were not visible. Compared to iodized oil, the enhancement of radiopaque microspheres was qualitatively comparable, but with lower intensity. However, the vascular distribution on histopathology was comparable between radiopaque and standard microspheres, as the sinusoidal penetration depth was not as deep as with iodized oil. The degree of inflammatory response was mild to moderate for radiopaque microspheres and moderate to severe for iodized oil. Different suspensions of the radiopaque microspheres resulted in different degrees of necrosis.

Radiopacity of embolic agents is potentially beneficial [6, 27, 28]. The intratumoral vascular distribution of the embolic agent determined by peri- and post-interventional imaging can be used for objective treatment control and to guide subsequent procedures, such as repeated embolization or thermal ablation procedures [6]. Furthermore, as already shown for iodized oil, non-enhancing areas may be an imaging biomarker of viable tumor or ineffective treatments [27]. Suk et al. demonstrated that contrast saturation in the tumor periphery can have a high positive predictive value for tumor response [28]. In the clinical setting, after embolization with standard microspheres, such imaging features can be achieved only with a low degree of reproducibility since the iodinated contrast agent is rapidly and unpredictably washed out [13, 24, 25]. Moreover, the transient intratumoral contrast agent saturation may not represent the exact location of the microspheres but mainly stasis of the iodinated contrast agent column [12, 13, 24, 29]. Using inherently radiopaque microspheres, the true intra- and peritumoral microsphere distribution could be assessed objectively, and treatment endpoints could be optimized and standardized (e.g. by using microsphere quantification techniques for the embolized target volume) [12, 13, 30]. Another important issue is the sensitivity of the imaging method which is being used for visualizing radiopaque embolic agents. In previous animal studies, the visibility of embolic agents was frequently assessed by applying experimental imaging techniques (e.g., micro CT or high-dose CT) [12, 14, 31]. For usability in clinical practice, the embolic agent needs to be visible when applying standard CT with clinically established acquisition and image reconstruction parameters.

In 2016, the first inherently radiopaque microspheres were approved by the US Food and Drug Administration (LC Bead LUMI [BTG, London, Great Britain]) [23]. LC Bead LUMI is a product of the modification of commercially available and established non-inherently radiopaque microspheres (LC Bead [BTG, London, Great Britain]) by incorporating iodized oil into the microspheres [13, 15]. Currently, the smallest commercially available inherently radiopaque microspheres are the above-mentioned LC Bead LUMI (version M1), which have a diameter of 75–150 μm. Even though these microspheres are considered to be small, theoretically they can be too large for deep penetration into the liver and thus ineffective for homogeneous occlusion of both the smallest tumor-feeding arteries and the vascular network of the tumor itself [7, 32, 33]. Therefore, inherently radiopaque microspheres with a smaller diameter and narrow-size calibration may be advantageous for effective tumor embolization [7, 34].

In this study, we demonstrated that microspheres with a mean diameter of 40 μm and a size range of 30–50 μm (95% confidence interval) occlude subsegmental arteries but can also reach interlobular arteries, and even sinusoids. Iodized oil droplets with a diameter of 10–50 μm exhibit deeper penetration into the liver with occlusion of mainly interlobular arteries and sinusoids. The higher intensity of arterial and parenchymal enhancement provided by iodized oil may be attributed to a greater amount and/or higher packing density of the radiopaque component, especially within interlobular arteries and sinusoids. For radiopaque microspheres, the suspension with the addition of iodinated contrast material (study group RMS.sa) exhibited a higher intensity of enhancement 2 hours after embolization, which can be explained by the iodinated contrast material itself and the temporary diffusion of iodinated contrast material into the microsphere core. On the other hand, both arterial and parenchymal enhancement diminishing during the 7 days of follow-up is most likely explained by a combination of the iodinated contrast material being washed out and microsphere redistributions.

The manufacturer of Embozene 40 warns not to include heparinized saline in the embolic mixture as this could lead to microsphere agglomeration. Microsphere agglomeration is unwanted as it may impede microsphere delivery through the catheter (e.g. occlusion) or result in uncontrolled embolization (e.g. non-target embolization or inhomogeneous microsphere distribution in the target volume). In this study, we did not observe any technical failures during embolization or adverse events after embolization for all study groups. However, we observed a higher degree of necrosis after microsphere embolization when heparin was added to the embolic agent (study group RMS-HEP.sa). In this context, it is important to mention the role of heparin in the reduction of coagulation thrombus as one mechanism of temporary arterial occlusion during embolization with microspheres. The higher degree of necrosis can be explained by more effective embolization by heparin-induced reduction of coagulation thrombus formation during embolization, increasing the packing density of the microspheres and reducing the potential for recanalization during follow-up. Embolization-induced necrosis was also observed by Sharma et al. after transarterial embolization without the addition of heparin in a pig liver model using LC BEAD LUMI microspheres 75–150 μm in size [14].

With respect to embolization-induced inflammation, a mild to moderate inflammatory response was observed for radiopaque microspheres, whereas a moderate to severe inflammatory response was observed for iodized oil. This finding has to be kept in mind because any inflammatory response can trigger an entire cytokine cascade, including neoangiogenesis, which may have implications for both tumor response and growth [35]. The mild to moderate inflammatory response for the radiopaque microspheres can be interpreted as acceptable biocompatibility [33, 36, 37].

This study has limitations. First, the number of animals was small and the specific embolization technique was designed for a proof of principle study. Therefore, a descriptive and comparative statistical analysis (e.g. measurement of HU) was consciously not performed. Second, there was no subacute setting (7 days survival) for the standard microsphere group. Third, 7 days was a short survival time for the subacute setting. Longer follow-up could provide more information on long-term visibility, tissue necrosis, and biocompatibility. Fourth, healthy pig livers were used in this study. The findings could be different for human liver tumors in regards to tumor biology, tumor microenvironment, and the complex tumor vascularity.

## Conclusion

Radiopaque 40μm-microspheres are visible on standard CT with qualitatively similar but quantitatively less intense enhancement compared to iodized oil. These microspheres also demonstrate less of an inflammatory reaction than iodized oil, but result in tissue necrosis, which was not observed after embolization with iodized oil. Both radiopaque and standard 40μm-microspheres are found within subsegmental and interlobar arteries, as well as in hepatic sinusoids.

## References

[1] Rammohan A, Sathyanesan J, Ramaswami S, Lakshmanan A, Senthil-Kumar P, Srinivasan UP, et al Embolization of liver tumors: Past, present and future. World journal of radiology. 2012;4(9):405–12. doi: 10.4329/wjr.v4.i9.405 ; PubMed Central PMCID: PMC3460228. 23024842 10.4329/wjr.v4.i9.405 PMC3460228

[2] Massmann A, Rodt T, Marquardt S, Seidel R, Thomas K, Wacker F, et al Transarterial chemoembolization (TACE) for colorectal liver metastases—current status and critical review. Langenbeck's archives of surgery. 2015;400(6):641–59. doi: 10.1007/s00423-015-1308-9 . 26088872 10.1007/s00423-015-1308-9

[3] Osuga K, Maeda N, Higashihara H, Hori S, Nakazawa T, Tanaka K, et al Current status of embolic agents for liver tumor embolization. International journal of clinical oncology. 2012;17(4):306–15. doi: 10.1007/s10147-012-0445-1 . 22806426 10.1007/s10147-012-0445-1

[4] Brown KT, Do RK, Gonen M, Covey AM, Getrajdman GI, Sofocleous CT, et al Randomized Trial of Hepatic Artery Embolization for Hepatocellular Carcinoma Using Doxorubicin-Eluting Microspheres Compared With Embolization With Microspheres Alone. J Clin Oncol. 2016;34(17):2046–53. doi: 10.1200/JCO.2015.64.0821 ; PubMed Central PMCID: PMCPMC4966514. 26834067 10.1200/JCO.2015.64.0821 PMC4966514

[5] Boulin M, Guiu B. Chemoembolization or Bland Embolization for Hepatocellular Carcinoma: The Question Is Still Unanswered. J Clin Oncol. 2017;35(2):256–7. doi: 10.1200/JCO.2016.67.2915 . 28056196 10.1200/JCO.2016.67.2915

[6] Sommer CM, Pallwein-Prettner L, Vollherbst DF, Seidel R, Rieder C, Radeleff BA, et al Transarterial embolization (TAE) as add-on to percutaneous radiofrequency ablation (RFA) for the treatment of renal tumors: Review of the literature, overview of state-of-the-art embolization materials and further perspective of advanced image-guided tumor ablation. Eur J Radiol. 2017;86:143–62. doi: 10.1016/j.ejrad.2016.10.024 . 28027741 10.1016/j.ejrad.2016.10.024

[7] Stampfl S, Bellemann N, Stampfl U, Sommer CM, Thierjung H, Lopez-Benitez R, et al Arterial distribution characteristics of Embozene particles and comparison with other spherical embolic agents in the porcine acute embolization model. Journal of vascular and interventional radiology: JVIR. 2009;20(12):1597–607. doi: 10.1016/j.jvir.2009.08.018 . 19944985 10.1016/j.jvir.2009.08.018

[8] Bonomo G, Pedicini V, Monfardini L, Della Vigna P, Poretti D, Orgera G, et al Bland embolization in patients with unresectable hepatocellular carcinoma using precise, tightly size-calibrated, anti-inflammatory microparticles: first clinical experience and one-year follow-up. Cardiovasc Intervent Radiol. 2010;33(3):552–9. doi: 10.1007/s00270-009-9752-y . 19957182 10.1007/s00270-009-9752-y

[9] Brown KT. Fatal pulmonary complications after arterial embolization with 40-120- micro m tris-acryl gelatin microspheres. Journal of vascular and interventional radiology: JVIR. 2004;15(2 Pt 1):197–200. . 14963189 10.1097/01.rvi.0000109400.52762.1f

[10] Gnutzmann DM, Mechel J, Schmitz A, Kohler K, Krone D, Bellemann N, et al Evaluation of the plasmatic and parenchymal elution kinetics of two different irinotecan-loaded drug-eluting embolics in a pig model. J Vasc Interv Radiol. 2015;26(5):746–54. Epub 2015/02/24. doi: 10.1016/j.jvir.2014.12.016 . 25704223 10.1016/j.jvir.2014.12.016

[11] Gockner TL, Zelzer S, Mokry T, Gnutzmann D, Bellemann N, Mogler C, et al Sphere-enhanced microwave ablation (sMWA) versus bland microwave ablation (bMWA): technical parameters, specific CT 3D rendering and histopathology. Cardiovasc Intervent Radiol. 2015;38(2):442–52. doi: 10.1007/s00270-014-0964-4 . 25167958 10.1007/s00270-014-0964-4

[12] Tacher V, Duran R, Lin M, Sohn JH, Sharma KV, Wang Z, et al Multimodality Imaging of Ethiodized Oil-loaded Radiopaque Microspheres during Transarterial Embolization of Rabbits with VX2 Liver Tumors. Radiology. 2016;279(3):741–53. doi: 10.1148/radiol.2015141624 ; PubMed Central PMCID: PMCPMC4886703. 26678453 10.1148/radiol.2015141624 PMC4886703

[13] Duran R, Sharma K, Dreher MR, Ashrafi K, Mirpour S, Lin M, et al A Novel Inherently Radiopaque Bead for Transarterial Embolization to Treat Liver Cancer—A Pre-clinical Study. Theranostics. 2016;6(1):28–39. Epub 2016/01/02. doi: 10.7150/thno.13137 ; PubMed Central PMCID: PMCPMC4679352. 26722371 10.7150/thno.13137 PMC4679352

[14] Sharma KV, Bascal Z, Kilpatrick H, Ashrafi K, Willis SL, Dreher MR, et al Long-term biocompatibility, imaging appearance and tissue effects associated with delivery of a novel radiopaque embolization bead for image-guided therapy. Biomaterials. 2016;103:293–304. doi: 10.1016/j.biomaterials.2016.06.064 . 27419364 10.1016/j.biomaterials.2016.06.064

[15] Negussie AH, Dreher MR, Johnson CG, Tang Y, Lewis AL, Storm G, et al Synthesis and characterization of image-able polyvinyl alcohol microspheres for image-guided chemoembolization. Journal of materials science Materials in medicine. 2015;26(6):198 doi: 10.1007/s10856-015-5530-3 . 26105830 10.1007/s10856-015-5530-3 PMC6663481

[16] Dreher MR, Sharma KV, Woods DL, Reddy G, Tang Y, Pritchard WF, et al Radiopaque drug-eluting beads for transcatheter embolotherapy: experimental study of drug penetration and coverage in swine. Journal of vascular and interventional radiology: JVIR. 2012;23(2):257–64 e4. doi: 10.1016/j.jvir.2011.10.019 ; PubMed Central PMCID: PMC3360470. 22178039 10.1016/j.jvir.2011.10.019 PMC3360470

[17] Imai N, Ishigami M, Ishizu Y, Kuzuya T, Honda T, Hayashi K, et al Transarterial chemoembolization for hepatocellular carcinoma: A review of techniques. World J Hepatol. 2014;6(12):844–50. doi: 10.4254/wjh.v6.i12.844 ; PubMed Central PMCID: PMCPMC4269903. 25544871 10.4254/wjh.v6.i12.844 PMC4269903

[18] de Baere T, Arai Y, Lencioni R, Geschwind JF, Rilling W, Salem R, et al Treatment of Liver Tumors with Lipiodol TACE: Technical Recommendations from Experts Opinion. Cardiovasc Intervent Radiol. 2016;39(3):334–43. doi: 10.1007/s00270-015-1208-y . 26390875 10.1007/s00270-015-1208-y

[19] Sommer CM, Kortes N, Mogler C, Bellemann N, Holzschuh M, Arnegger F, et al Super-micro-bland particle embolization combined with RF-ablation: angiographic, macroscopic and microscopic features in porcine kidneys. Eur J Radiol. 2012;81(6):1165–72. doi: 10.1016/j.ejrad.2011.03.022 . 21458182 10.1016/j.ejrad.2011.03.022

[20] Stampfl U, Sommer CM, Bellemann N, Holzschuh M, Kueller A, Bluemmel J, et al Multimodal visibility of a modified polyzene-F-coated spherical embolic agent for liver embolization: feasibility study in a porcine model. J Vasc Interv Radiol. 2012;23(9):1225–31 e2. doi: 10.1016/j.jvir.2012.06.008 . 22832143 10.1016/j.jvir.2012.06.008

[21] Sommer CM, Stampfl U, Bellemann N, Holzschuh M, Kueller A, Bluemmel J, et al Multimodal visibility (radiography, computed tomography, and magnetic resonance imaging) of microspheres for transarterial embolization tested in porcine kidneys. Investigative radiology. 2013;48(4):213–22. doi: 10.1097/RLI.0b013e31827f6598 . 23399807 10.1097/RLI.0b013e31827f6598

[22] Lanza E, Donadon M, Poretti D, Pedicini V, Tramarin M, Roncalli M, et al Transarterial Therapies for Hepatocellular Carcinoma. Liver Cancer. 2016;6(1):27–33. doi: 10.1159/000449347 ; PubMed Central PMCID: PMCPMC5159740. 27995085 10.1159/000449347 PMC5159740

[23] Levy EB, Krishnasamy VP, Lewis AL, Willis S, Macfarlane C, Anderson V, et al First Human Experience with Directly Image-able Iodinated Embolization Microbeads. Cardiovasc Intervent Radiol. 2016;39(8):1177–86. doi: 10.1007/s00270-016-1364-8 . 27206503 10.1007/s00270-016-1364-8 PMC7831151

[24] Golowa YS, Cynamon J, Reinus JF, Kinkhabwala M, Abrams M, Jagust M, et al Value of noncontrast CT immediately after transarterial chemoembolization of hepatocellular carcinoma with drug-eluting beads. Journal of vascular and interventional radiology: JVIR. 2012;23(8):1031–5. doi: 10.1016/j.jvir.2012.04.020 . 22739645 10.1016/j.jvir.2012.04.020

[25] Wang X, Erinjeri JP, Jia X, Gonen M, Brown KT, Sofocleous CT, et al Pattern of retained contrast on immediate postprocedure computed tomography (CT) after particle embolization of liver tumors predicts subsequent treatment response. Cardiovascular and interventional radiology. 2013;36(4):1030–8. doi: 10.1007/s00270-012-0506-x ; PubMed Central PMCID: PMC4394653. 23152036 10.1007/s00270-012-0506-x PMC4394653

[26] Iwai K, Maeda H, Konno T. Use of oily contrast medium for selective drug targeting to tumor: enhanced therapeutic effect and X-ray image. Cancer Res. 1984;44(5):2115–21. . 6324996

[27] Takayasu K, Arii S, Matsuo N, Yoshikawa M, Ryu M, Takasaki K, et al Comparison of CT findings with resected specimens after chemoembolization with iodized oil for hepatocellular carcinoma. AJR Am J Roentgenol. 2000;175(3):699–704. doi: 10.2214/ajr.175.3.1750699 . 10954453 10.2214/ajr.175.3.1750699

[28] Suk Oh J, Jong Chun H, Gil Choi B, Giu Lee H. Transarterial chemoembolization with drug-eluting beads in hepatocellular carcinoma: usefulness of contrast saturation features on cone-beam computed tomography imaging for predicting short-term tumor response. Journal of vascular and interventional radiology: JVIR. 2013;24(4):483–9. doi: 10.1016/j.jvir.2013.01.001 . 23452553 10.1016/j.jvir.2013.01.001

[29] Loffroy R, Lin M, Yenokyan G, Rao PP, Bhagat N, Noordhoek N, et al Intraprocedural C-arm dual-phase cone-beam CT: can it be used to predict short-term response to TACE with drug-eluting beads in patients with hepatocellular carcinoma? Radiology. 2013;266(2):636–48. doi: 10.1148/radiol.12112316 ; PubMed Central PMCID: PMC3558876. 23143027 10.1148/radiol.12112316 PMC3558876

[30] Aliberti C, Carandina R, Sarti D, Pizzirani E, Ramondo G, Cillo U, et al Transarterial chemoembolization with DC Bead LUMI radiopaque beads for primary liver cancer treatment: preliminary experience. Future oncology. 2017;13(25):2243–52. doi: 10.2217/fon-2017-0364 . 29063780 10.2217/fon-2017-0364

[31] Sharma KV, Dreher MR, Tang Y, Pritchard W, Chiesa OA, Karanian J, et al Development of "imageable" beads for transcatheter embolotherapy. J Vasc Interv Radiol. 2010;21(6):865–76. doi: 10.1016/j.jvir.2010.02.031 ; PubMed Central PMCID: PMCPMC2876341. 20494290 10.1016/j.jvir.2010.02.031 PMC2876341

[32] Verret V, Ghegediban SH, Wassef M, Pelage JP, Golzarian J, Laurent A. The arterial distribution of Embozene and Embosphere microspheres in sheep kidney and uterus embolization models. J Vasc Interv Radiol. 2011;22(2):220–8. doi: 10.1016/j.jvir.2010.10.021 . 21276915 10.1016/j.jvir.2010.10.021

[33] Stampfl S, Stampfl U, Rehnitz C, Schnabel P, Satzl S, Christoph P, et al Experimental evaluation of early and long-term effects of microparticle embolization in two different mini-pig models. Part II: liver. Cardiovasc Intervent Radiol. 2007;30(3):462–8. doi: 10.1007/s00270-005-0350-3 . 17342551 10.1007/s00270-005-0350-3

[34] Wang YX, De Baere T, Idee JM, Ballet S. Transcatheter embolization therapy in liver cancer: an update of clinical evidences. Chin J Cancer Res. 2015;27(2):96–121. doi: 10.3978/j.issn.1000-9604.2015.03.03 ; PubMed Central PMCID: PMCPMC4409973. 25937772 10.3978/j.issn.1000-9604.2015.03.03 PMC4409973

[35] Sergio A, Cristofori C, Cardin R, Pivetta G, Ragazzi R, Baldan A, et al Transcatheter arterial chemoembolization (TACE) in hepatocellular carcinoma (HCC): the role of angiogenesis and invasiveness. Am J Gastroenterol. 2008;103(4):914–21. doi: 10.1111/j.1572-0241.2007.01712.x . 18177453 10.1111/j.1572-0241.2007.01712.x

[36] Stampfl S, Stampfl U, Bellemann N, Sommer CM, Thierjung H, Radeleff B, et al Biocompatibility and recanalization characteristics of hydrogel microspheres with polyzene-F as polymer coating. Cardiovasc Intervent Radiol. 2008;31(4):799–806. doi: 10.1007/s00270-007-9268-2 . 18214594 10.1007/s00270-007-9268-2

[37] Stampfl S, Stampfl U, Rehnitz C, Schnabel P, Satzl S, Christoph P, et al Experimental evaluation of early and long-term effects of microparticle embolization in two different mini-pig models. Part I: kidney. Cardiovasc Intervent Radiol. 2007;30(2):257–67. doi: 10.1007/s00270-005-0309-4 . 17216380 10.1007/s00270-005-0309-4

